# Solubility Study of Acetylsalicylic Acid in Ethanol + Water Mixtures: Measurement, Mathematical Modeling, and Stability Discussion

**DOI:** 10.1208/s12249-021-02192-7

**Published:** 2021-12-28

**Authors:** Ali Nokhodchi, Taravat Ghafourian, Nour Nashed, Kofi Asare-Addo, Elmira Behboudi, Yasaman Sefid-Sefidehkhan, Aynaz Zarghampour, Elaheh Rahimpour, Abolghasem Jouyban

**Affiliations:** 1 School of Life Sciences, University of Sussex, Brighton, BN1 9QJ UK; 2 School of Life Sciences, Faculty of Creative Arts, Technologies and Science, University of Bedfordshire, Luton, UK; 3 Department of Pharmacy, University of Huddersfield, Huddersfield, UK; 4 Drug Applied Research Center, Tabriz University of Medical Sciences, Tabriz, Iran; 5 Department of Physical Chemistry, Faculty of Chemistry, University of Tabriz, Tabriz, Iran; 6 Department of Chemistry, University of Mohaghegh Ardabili, Ardabil, Iran; 7 Student Research Committee, Faculty of Pharmacy, Tabriz University of Medical Sciences, Tabriz, Iran; 8 Pharmaceutical Analysis Research Center and Faculty of Pharmacy, Tabriz University of Medical Sciences, Tabriz, Iran; 9 Food and Drug Safety Research Center, Tabriz University of Medical Sciences, Tabriz, Iran; 10 Faculty of Pharmacy, Near East University, Box 99138 Nicosia, North Cyprus, 10 Mersin, PO Turkey

**Keywords:** acetylsalicylic acid, solubility prediction, thermal analysis, ethanol, PC-SAFT EOS

## Abstract

Solubility determination of poorly water-soluble drugs is pivotal for formulation scientists when they want to develop a liquid formulation. Performing such a test with different ratios of cosolvents with water is time-consuming and costly. The scarcity of solubility data for poorly water-soluble drugs increases the importance of developing correlation and prediction equations for these mixtures. Therefore, the aim of the current research is to determine the solubility of acetylsalicylic acid in binary mixtures of ethanol+water at 25 and 37°C. Acetylsalicylic acid is non-stable in aqueous solutions and readily hydrolyze to salicylic acid. So, the solubility of acetylsalicylic acid is measured in ethanolic mixtures by HPLC to follow the concentration of produced salicylic acid as well. Moreover, the solubility of acetylsalicylic acid is modeled using different cosolvency equations. The measured solubility data were also predicted using PC-SAFT EOS model. DSC results ruled out any changes in the polymorphic form of acetylsalicylic acid after the solubility test, whereas XRPD results showed some changes in crystallinity of the precipitated acetylsalicylic acid after the solubility test. Fitting the solubility data to the different cosolvency models showed that the mean relative deviation percentage for the Jouyban-Acree model was less than 10.0% showing that this equation is able to obtain accurate solubility data for acetylsalicylic acid in mixtures of ethanol and water. Also, the predicted data with an average mean relative deviation percentage (***MRD***%) of less than 29.65% show the capability of the PC-SAFT model for predicting solubility data. A brief comparison of the solubilities of structurally related solutes to acetylsalicylic acid was also provided.

## INTRODUCTION

It has been reported that non-steroidal anti-inflammatory drugs are among the most commonly used drugs (1). Based on the biopharmaceutics classification system (BCS) proposed by Amidon *et al.*(2), these drugs belong to class II, characterized by low solubility and high permeability (3). Therefore, dissolution/solubility plays a key role in better absorption and the fast dissolution of BCS class II drugs in the gastrointestinal tract (GIT) following oral administration (4). Because of high membrane permeability, the degree of absorption of these drugs could have been as high as 100% if they did not suffer poor solubility/dissolution in the GIT. However, this may be difficult because the drugs display poor aqueous solubility (5). Poor aqueous solubility could be a result of high lipophilicity and strong intermolecular interactions which make the solubilization of the solid energetically costly (6).

The solubility of a drug in the GIT is one of the main parameters in formulating a drug into a dosage form as it can control bioavailability. So, the poor solubility of a drug in the GIT can present a key issue during drug formulation development. Furthermore, the solubility of drugs in water cannot be ignored in the formulation development of liquid, parenteral formulations, and soft gelatine capsules. In the case of poorly water-soluble drugs, the solubility should be enhanced to enable the formulator to develop liquid dosage forms. This is the main reason that researchers are interested in exploring the solubility profile of a specific drug in a specific solvent as it permits the scientists to select the ideal solvent medium for drug formulation. In addition, the solubility knowledge can help overcome problems arising in the re-crystallization of drugs by changing solvent polarity and/or temperature of the solution. In addition, cosolvency has wider application throughout the chemical industry. It has been used for soil remediation to increase the solubility of contaminants in water for easier removal of toxic contaminations such as pesticides (7).

Aqueous solubility of acetylsalicylic acid at 278.15–345.15 K was reported by Apelblat and Manzurola (8). The enhancement effects of a number of surfactants on the aqueous solubility of acetylsalicylic acid at 37°C (9) and also the solubility of acetylsalicylic acid in a number of mono-solvents at various temperatures were reported (10, 11). Effects of four hydrotropes on the solubilization of acetylsalicylic acid at different temperatures were also investigated (12).

The calculation of drug solubility in binary mixtures of solvents is paramount important as such the knowledge could provide useful information to researchers to find the binary solvents which are capable of dissolving more drugs. Generally, in the formulation development of liquid dosage forms, the optimum concentration of the cosolvent in water is normally determined by trial and error experimentations. But this approach not only is time-consuming but also is a costly process. To overcome these two drawbacks, cosolvency data can be modeled for prediction purposes. Such cosolvency models often predict a given drug’s solubility in various fractions of a single cosolvent-water mixture based on the known solubility of the drug in each of the two neat solvents and solubility in several cosolvent-water fractions (13). Examples of these cosolvency models are the extended Hildebrand solubility approach (EHS)(14), the log-linear model of Yalkowsky (15), the excess free energy approach (16), the Jouyban-Acree model (17) as an extended version of the combined nearly ideal binary solvent/Redlich-Kister equation (CNIBS/R-K) (18), the general single model (GSM)(19), and the mixture response surface methods (MR-S)(20). Although the aforementioned cosolvency models are very useful, some of them lack generalization to other drugs’ solubility in the same cosolvent-water mixtures, or extrapolation to other cosolvents, when the solubility of the drug in the neat solvents and the solubilizing power of the cosolvents are not known.

There have been efforts to predict cosolvency of various solvents by using physicochemical properties of the solvents (21, 22), which allow prediction of a given drug’s solubility in various cosolvent-water mixtures. Moreover, the solubilization profiles of a set of cosolvents (mixed with water) towards a group of chemically unrelated drugs have been shown to differ according to the hydrophobicity of the solutes (23) or Abraham parameters of the solvents and drug (24). By employing the quantitative structure-property relationship (QSPR), it is possible to estimate the solubility of drugs from the properties of the molecular structures of the drugs. Comparison of QSPR models for drug solubility in different solvents can identify solute features determining solubility in various solvents or solvent mixtures. One such investigation by Ghafourian and Bozorgi (25) has shown that the impact of drug hydrophobicity (log P) on solubility is reduced at higher volume fractions of PEG in the PEG/water binary mixtures.

In addition to the success of predicting solubility data with the mentioned models, researchers have recently paid special attention to the perturbed-chain statistical associating fluid theory (PC-SAFT) model. The main advantage of the PC-SAFT model is that it uses only data from quantum chemical calculations, thus enabling predictions when there are no experimental data available. Until now, the drug solubility in the different solutions such as molecular solvents, CO_2_, ionic liquids, and deep eutectic solvents has been modeled using PC-SAFT (26–30). However, such studies are few and mostly considered the simplified models or cubic equation of states (EOSs)(25). In this study, using the PC-SAFT EOS, the solubility of acetylsalicylic acid in the presence of binary mixtures of ethanol + water at 25 and 37°C was estimated. Acetylsalicylic acid is non-stable in aqueous solutions and readily hydrolyzes to salicylic acid (31–34). The hydrolysis rate could be decreased by the addition of polyethylene glycol (PEG) 6000, povidone, or sorbitol (35). The half-life of the hydrolysis of acetylsalicylic acid at 22.5°C in unbuffered water is 153.30 h, PEG 400 + water (ratio 1:4) is 359.80 h, phosphate buffer pH 7.0 is 75.30 h, phosphate buffer pH 7.4 is 82.40 h, and phosphate buffer pH 7.4 at 37°C is 15.40 h (34). These values for 1,4-dioxane, acetonitrile, tetrahydrofuran, propan-2-ol, water, methanol, and ethanol at 21°C were reported as 83.35, 63.37, 62.14, 41.43, 38.09, 8.83, and 8.15 h, respectively (33). It has also been shown that the degradation rate of acetylsalicylic acid in ethanol + water mixtures is increased by increasing ethanol fraction and temperature (31). Concerning this information, the determination of acetylsalicylic acid solubility in ethanol + water mixtures is a challenging topic and needs further consideration. The aim of the current study was to use acetylsalicylic acid as a non-stable model drug and water-ethanol as safe solvents to use in pharmaceutical formulation to calculate the solubility of the drug with a minimal error.

## MATERIALS AND METHODS

Acetylsalicylic acid (ASA or aspirin) powder with a mass fraction purity of >99% was obtained from Sigma (Sigma-Aldrich, Gillingham, UK). Absolute ethanol was purchased from Fisher Scientific (Loughborough, UK).

### Solubility Studies

Eleven combinations of binary solvent mixtures were prepared using suitable volume fractions of water-ethanol increasing consecutively from 0.0 to 1.0 ethanol: water, the neat solvents. A shake-flask technique with spectrophotometry was used to determine drug solubility in these solvents. Excess amounts of drug were added to the mixtures to allow saturation concentration to be reached. The eleven samples were then incubated at 37°C and 25°C in a thermostatic water bath shaker (Cambridge, Crafton) at atmospheric pressure while constantly shaking at a speed of 200 rpm, for a minimum of 72 h (the preliminary results showed that 72 h was sufficient to reach equilibrium condition).

The suspensions were allowed to settle for 1 h, and then the supernatants were filtered to remove the excess solid using a syringe-driven filter unit (pore size 0.20 μm). A volume of either 0.2 mL or 1 mL (depending on the concentration of drug in the saturated sample) of the filtrate from each sample was immediately diluted quantitatively using an appropriate amount of the same ethanol: water solvent mixture. Dilutions in the range of 10–10,000 times were made depending on the concentration of the drug in the filtered solutions. A Knauer HPLC instrument (Berlin, Germany) composed of a K-1001 HPLC pump, a BioTech. degasser, a sample loop (20 μL), and a K-2600 ultraviolet detector was used for the determination of acetylsalicylic acid and salicylic acid. The chromatographic data processing was performed by employing the Chromgate software (version 3.1). The separation was performed by a stationary phase of C_18_ XBridge analytical column (5 μm × 250 mm × 4.6 mm) from Waters Co. (Ireland), and the mobile phase consisted of phosphoric acid:acetonitrile:water (2:400:600 v/v). The freshly prepared mobile phase was filtered using a vacuum filter system equipped with a 0.45-μm membrane filter (Millipore Corp., Billerica, Massachusetts) and degassed by ultrasonic for 15 min. Chromatography was run at 25°C by pumping the mobile phase at a flow rate of 1.5 mL/min. The UV detector recorded the column effluent at 254 nm (36). The calibration curve between the peak area and the concentration in the range of 10–1000 mg.L^−1^ for acetylsalicylic acid is *Y* = 3336.8 C_ASA_ + 181,813 and in the range of 0.5–20 mg.L^−1^ for salicylic acid is *Y* = 39,158 C_SA_ + 14,652. The solubility data are obtained from the interpolation of these plots.

### Density Measurement of the Solutions

In order to measure the true density of each saturated solution, 1 mL of each filtrate was weighed accurately using an accurate balance with a precision of 0.1 mg (Sartorius, Ireland). The true density values are needed to convert molarity and mole fraction. Three repeats of the procedure were completed for all seven drugs at 37°C and 25°C, and the results averaged. It should be noted that the density of the saturated solution of acetylsalicylic acid is overestimated since there is a considerable amount of salicylic acid which increases the density value.

### Differential Scanning Calorimetry

A differential scanning calorimeter (DSC7, Mettler Toledo, Switzerland) was employed to investigate the thermal behavior (enthalpy and melting point) of acetylsalicylic acid before and after the solubility test. This information allows us to identify whether a different polymorphic form is produced during the solubility test. The samples studied through the DSC machine were the pure drug sample and the samples obtained after equilibration with 0, 0.5, and 1 ethanol fractions at both 25 and 37°C temperatures. The acetylsalicylic acid particles left in the solubility test were collected and dried. The dried samples were placed in DSC pans and heated between 25 and 300°C at a scanning rate of 10°C/min under nitrogen gas (50 mL/min). After obtaining the DSC traces for each sample, the melting points and enthalpies of fusion were calculated by the software provided.

### Fourier Transform Infrared Spectroscopy

In order to explore any changes in the structure of the solid extracted from the solubility test as a result of possible hydrolysis of acetylsalicylic acid and precipitation of any by-products, FT-IR was employed (Perkin Elmer’s Spectrum One, Shelton, CT, USA). Briefly, methanol was used to clean the instrument to remove any residual matter left on the apparatus, followed by placing a few milligrams of each of the separated solid particles after the solubility test. The sample was pressed with a pressure of 100 bar followed by scanning the sample three times over a range of 4000 cm^−1^ to 500 cm^−1^ to obtain spectra with appropriate resolution.

### X-ray Powder Diffraction

The XRPD patterns were obtained for all samples including original acetylsalicylic acid using a D2 Phaser diffractometer (Bruker AXS GmbH, Karlsruhe, Germany). All samples produced were scanned in Bragg-Brentano geometry, over a scattering (Bragg, 2θ) angle range from 5 to 50°, in 0.02° steps at 1.5° min^−1^(37). Microsoft Excel was used to analyze and plot the collected XRPD patterns. The crystallinity of the samples was also determined to elucidate the effect of the type of solvent (water and ethanol) on the crystallinity of the recovered acetylsalicylic acid samples. The area under the curve for the “distinctive crystalline peaks” at 7.8 and 15.6 2θ angles was measured for each XRPD diffractogram and used in the determination of crystallinity (%) using the equation 1(38).

1 $$\mathrm{Crystallinity}=\frac{\mathrm{Area}\ \mathrm{of}\ \mathrm{cryatlline}\ \mathrm{peaks}}{\mathrm{Area}\ \mathrm{of}\ \mathrm{all}\ \mathrm{peaks}\ \left(\mathrm{crystalline}+\mathrm{amorphous}\right)}\times 100$$

### Mathematical Modeling of the Solubility Data

The solubility values determined for acetylsalicylic acid in ethanol + water mixtures are correlated and back-calculated utilizing the mathematical cosolvency models such as the Yalkowsky, Jouyban-Acree, modified Wilson, and PC-SAFT models, and details for each studied model are discussed below.

#### Yalkowsky Model

The Yalkowsky model was employed to express the natural logarithm of solubility in a mixture of solvent + cosolvent (39).

2 $$\ln {C}_m={f}_1\ln {C}_1+{f}_2\ln {C}_2$$

where *C*_*1*_ and *C*_*2*_ are solubility data in mono-solvents 1 and 2 in molar fraction unit, *x*_*m*_ is the solubility of the drug in the solvent mixture, and *f*_1_ and *f*_2_ are volume fractions of solvents 1 and 2 in the absence of the drug. After modification of Eq. 2 (i.e., substitution of *f*_*2*_ with (1*−f*_*1*_) and subsequent rearrangements), Eq. 3 can be obtained as (40):

3 $$\ln {C}_m=\ln {C}_2+\left(\ln \frac{C_1}{C_2}\right){f}_1=\ln {C}_2+\sigma .{f}_1$$

the *σ* is the model constant. A linear relationship has been shown between the logarithm of octanol-to-water partition coefficient of the solute (log *P*) and σ as below (41):

4 $$\sigma =M.\log P+N$$

where *M* and *N* are the cosolvent constants. After replacing Eq. 4 in Eq. 3, a predictive mathematical equation is attained (41).

5 $$\ln {C}_m=\ln {C}_2+{f}_1\left(M.\log P+N\right)$$

By employing *M* and *N* values obtained from the literature for used cosolvent (ethanol) and log *P* of a drug, the solubility of the drug in the solvent mixture can be computed only using solubility data in water.

#### Jouyban-Acree Model

The Jouyban-Acree model as a simple linear cosolvency model used for binary mixtures of solvents at various temperatures can be presented by Eq. 6(17):

6 $$\ln {C}_{m,T}={f}_1.\ln {C}_{1,T}+{f}_2.\ln {C}_{2,T}+\frac{f_1.{f}_2}{T}\sum_{i=0}^2{J}_i.{\left({f}_1-{f}_2\right)}^i$$

*J*_*i*_ is the model parameter calculated using linear regression of (ln*C*_*m*, *T*_ − *f*_1_. ln *C*_1, *T*_ − *f*_2_. ln *C*_2, *T*_) *vs*$\frac{f_1.{f}_2}{T}$, $\frac{f_1.{f}_2\left({f}_1-{f}_2\right)}{T}$, and $\frac{f_1.{f}_2{\left({f}_1-{f}_2\right)}^2}{T}$ and other model parameters have the same meanings as those of the above model.

#### The Modified Wilson Model

In addition to linear models employed for fitting and prediction of solubility values, the non-linear model of modified Wilson is also utilized for modeling the solubility data in the solvent mixtures at isothermal conditions. The equation is as (42):

7 $$-\ln {C}_m=1-\frac{f_1\left[1+\ln {x}_1\right]}{f_1+{f}_2{\lambda}_{12}}-\frac{f_2\left[1+\ln {x}_2\right]}{f_1{\lambda}_{21}+{f}_2}$$

*λ*_12_ and *λ*_21_ are the model constants computing using nonlinear analysis.

#### PC-SAFT Model

The perturbed chain SAFT equation of state (EOS) or PC-SAFT was first proposed and developed by Gross and Sadowski in 2001 (43) as an alternative to the original version of SAFT derived by Chapman *et al.*(44). The residual molar Helmholtz energy of the PC-SAFT (*a*^res^) obtained by the Helmholtz energy contributions from the reference system hard chain (*a*^hc^), dispersion force (*a*^***disp***^), and hydrogen bonding (*a*^***assoc***^) is obtained as follows:

8 $${a}^{\mathrm{res}}=a-{a}^{\boldsymbol{ideal}}={a}^{\mathrm{hc}}+{a}^{\boldsymbol{disp}}+{a}^{\boldsymbol{assoc}}$$

In PC-SAFT, pure components can be described using five pure-component parameters: (i)*m*, number of segments per chain; (ii)*σ*, diameter of each segment in Angstrom (Å); (iii)*ε*, energy parameter for each segment in Joules (J); (iv) *κ*^AiBi^, effective volume of the association (Å^3^); (v)*ε*^AiBi^, energy parameter of the association (bar.l/mol); (vi)*NumAss*, number of association sites (${N}_i^{\boldsymbol{assoc}}$). The parameters of each component are reported in Table I(43, 45). The interaction parameters for binary systems (ethanol + water), (acetylsalicylic acid + water) and (acetylsalicylic acid + ethanol), *k*_ij_, for the purely predictive model were set to zero.

**Table I:** Pure Component Parameters for the Substances

Chemical name	*m*_i_	*σ*(*A*^∘^)	*ε*/*k*(*K*)	${K}^{A_i{B}_j}$	${\varepsilon}^{A_i{B}_j}$	Associating scheme	Ref.
Acetylsalicylic acid	5.5830	3.8593	256.38	0.01	2453.80	2B	33
Ethanol	2.3827	3.1771	198.2	0.032384	2653.4	2B	31
Water	1.0656	3.0007	366.51	0.034868	2500.7	2B	31

The fugacity coefficient for component *k* (*ϕ*_*k*_) and compressibility factor (*z*) using the PC-SAFT EOS are computed as follows:

9 $$\mathit{\ln}{\phi}_k={a}^{\mathrm{res}}+\left(z-1\right)+{\left(\frac{\partial {a}^{\mathrm{res}}}{\partial {x}_k}\right)}_{T,V,{X}_{i\ne k}}-\sum_{j=1}^N\left[{X}_j{\left(\frac{\partial {a}^{\mathrm{res}}}{\partial {X}_k}\right)}_{T,V,{X}_{i\ne j}}\right]- lnz$$

10 $$z=1+\rho {\left(\frac{\partial {a}^{\mathrm{res}}}{\partial \rho}\right)}_{T,{X}_i}$$

where *ρ* is the molar density. Using PC-SAFT, the activity coefficients are calculated from the fugacity coefficients through Eq. 11:

11 $${\gamma}_i=\frac{\phi_i}{\phi_i^0}$$

where *ϕ*_*i*_ and ${\phi}_i^0$ are the fugacity coefficients of component *i* in the mixture and that of the pure compound, respectively. In solid-liquid equilibria, the solid solubility in the liquid phase is calculated according to the following expression (46):

12 $$\mathit{\ln}{x}_i=\frac{\Delta {H}_m}{R}\left(\frac{1}{T_m}-\frac{1}{T}\right)-\mathit{\ln}{\gamma}_i$$

where *x*_*i*_ and *γ*_*i*_ represent the solubility and activity coefficient of compound *i*. In this study, the activity coefficient of compound *i* (*γ*_*i*_) was determined via Eq. 11. Since the activity coefficient depends on solubility in mole fraction (*x*_*i*_), solubility must be determined from the iterations with Eq. 12. In the equation above, Δ*H*_*m*_ and *T*_*m*_ represent fusion enthalpy and melting point temperature, respectively, and their values are presented in Table II.

**Table II:** Melting Points and Enthalpy of Fusion for Drug Crystals Obtained From Different Concentrations of Ethanol + Water at 25 and 37°C

Sample (ethanol:water%)	Melting point (°C)		Enthalpy of fusion (J/g)	
	25°C	37°C	25°C	37°C
0:100	142.9±1.0	137.8±0.5	147.7±2.7	130.7±2.8
50:50	141.3±0.1	144.4±0.2	139.5±0.4	161.0±1.7
100:0	143.7±0.2	144.3±0.0	156.2±3.7	155.4±2.0
Untreated acetylsalicylic acid	145.3±0.1	145.3±0.1	165.6±6.2	165.6±6.2

All explained models are correlated to the measured solubility values of acetylsalicylic acid, and the mean relative deviation (*MRD*%) (Eq. 13) is used to obtain the model’s accuracy.

13 $$MRD\%=\frac{100}{N}\sum \left(\frac{\left|\boldsymbol{Calculatedvalue}-\boldsymbol{Observedvalue}\right|}{\boldsymbol{Observedvalue}}\right)$$

*N* is the number of data points in each set.

## RESULTS AND DISCUSSION

### Thermal Analysis

The melting point and enthalpy of fusion were determined for pure acetylsalicylic acid as well as for the samples that had undergone solubility experiments following treatment with 0%, 50%, and 100% ethanol. Since the solid phase remaining at the end of the solubility experiments could have altered to the different polymorphic forms as a result of the equilibrium state during the solubility experiments, the enthalpy and melting point of the solid particles obtained after each solubility test were compared to the original drug crystals. Acetylsalicylic acid has two polymorphic forms known as polymorph I and polymorph II. Polymorph I which is the stable form of acetylsalicylic acid melts above 137°C (47), whereas polymorph II melts between 128 and 130°C (48). The DSC traces showed that the concentration of ethanol + water had no effect on the type of polymorphic form of acetylsalicylic acid since the filtered acetylsalicylic acid after the solubility experiments showed a melting peak above 137°C. The results of Table II ruled out the presence of polymorph II in the acetylsalicylic acid samples as the melting points of all samples are above 137°C hence portraying the absence of changes in the polymorphic form of acetylsalicylic acid before and after the solubility test.

Although some authors suggested that the onset melting temperature of the solute is preferred over the peak melting temperature (49), others such as El-Badry*et al*. (50) employed the peak temperature to represent the melting point of the drugs. Likewise, this study utilized the peak melting temperature. On the whole, it can be observed that the melting points of the samples (pure drug and the samples from solubility experiments) were slightly different from each other. This could be due to changes in the particle size or the crystallinity of the original acetylsalicylic acid particles after the solubility test where some of the dissolved acetylsalicylic acid particles can undergo recrystallization leading to different particle sizes and different crystallinity percentages. It has been shown that, generally, smaller particles should melt at a lower temperature than when larger particles are used for the given material (51). This is true due to the high surface area of small particles that would be in contact with the heat during DSC hence lowering the melting point.

**Figure 1:**
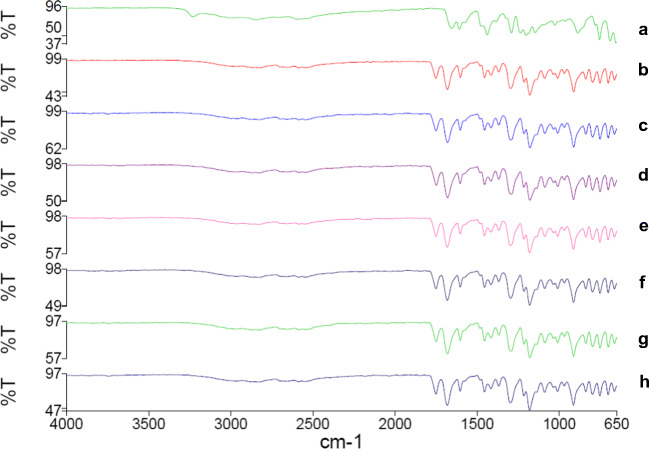
FT-IR spectra of all samples collected from each solubility test and original acetylsalicylic acid: (a) salicylic acid; (b) acetylsalicylic acid; acetylsalicylic acid collected at 37°C from (c) 100% water, (d) 100% ethanol, and (e) 50:50 water:ethanol; and acetylsalicylic acid collected at 25°C from (f) 100% water, (g) 100% ethanol, and (h) 50:50 water:ethanol

In order to explore any changes in the structure of acetylsalicylic acid crystals as a result of hydrolysis (degradation), FT-IR was carried out on the extracted acetylsalicylic acid after the solubility test. FT-IR spectra of all samples showed that all the diagnostic peaks for acetylsalicylic acid can be also detected in the samples collected after the solubility test at different temperatures (Figure 1). Furthermore, the FT-IR spectra of the collected samples after the solubility test did not show any peak relevant to the presence of salicylic acid. This indicates that salicylic acid is not reached above the saturated solubility to precipitate.

Table II shows that the enthalpy of fusion is different compared to the enthalpy of untreated acetylsalicylic acid. For example, the enthalpy of fusion of unprocessed acetylsalicylic acid was 165.6 ± 6.2 J/g, whereas this value was 130.7±2.8 J/g when 100% water was used in the solubility test. Bustamante *et al.*(52) suggested that the small differences in enthalpy of fusion values observed after contact with the solvents are not sufficiently important and thermodynamic activity can be assumed constant in the solubility model which could be true when 50:50 water:ethanol or 100% ethanol was used in the solubility test as their enthalpy is closer to the enthalpy of untreated acetylsalicylic acid. Table II shows that the enthalpy difference between unprocessed acetylsalicylic acid and when 100% water was used in the solubility test is significant. It has been reported that the significant difference in the enthalpy could be due to changes in the crystallinity of acetylsalicylic acid after the solubility test. This has been shown when acetylsalicylic acid has been crystallized from various organic solvents (53). The enthalpy data in Table II shows that the acetylsalicylic acid crystals filtered after the solubility test in 100% water have less crystallinity compared to untreated acetylsalicylic acid.

**Figure 2:**
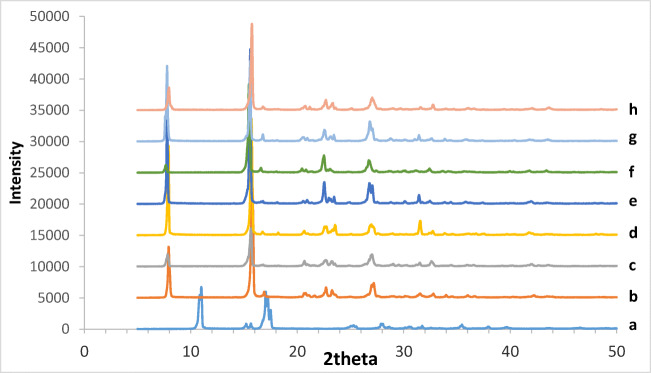
XRPD spectra of all samples collected from each solubility test and original acetylsalicylic acid: (a) salicylic acid; (b) acetylsalicylic acid; acetylsalicylic acid collected at 37°C from (c) 100% water, (d) 100% ethanol, and (e) 50:50 water:ethanol; and acetylsalicylic acid collected at 25°C from (f) 100% water, (g) 100% ethanol, and (h) 50:50 water:ethanol

**Figure 3:**
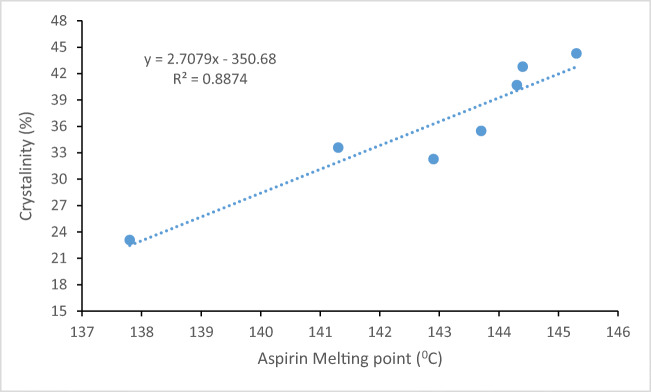
Relationship between melting point and crystallinity of the extracted acetylsalicylic acid after the solubility test

**Figure 4:**
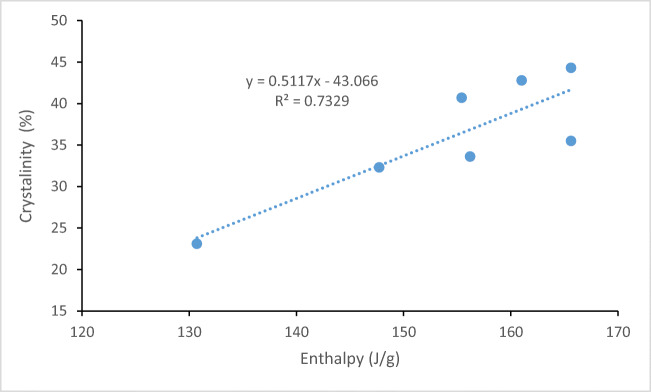
Relationship between enthalpy and crystallinity of the extracted acetylsalicylic acid after the solubility test

Since the crystallinity of acetylsalicylic acid could change the solubility, XRPD was used to investigate any changes in the crystallinity of the collected acetylsalicylic acid samples compared to the original acetylsalicylic acid after the solubility test (Figure 2). The XRPD of the samples shows that the only difference between the samples is the intensity of peaks at 7.8 and 15.6 2θ. The change in the intensity of the peaks could be due to changes in the crystallinity of the samples collected from different solvents at different temperatures. The relative crystallinity of the samples was calculated (Table III), and the results showed that the crystallinity of the samples collected at 25°C has not significantly changed (ranging from 32.3 to 35.5%), but this was not the case for the samples collected at 37°C. The crystallinity of the acetylsalicylic acid samples collected from 100% water was significantly lower than that of other samples which could increase the solubility of acetylsalicylic acid during the solubility test. This is in agreement with melting point and enthalpy data (Table II) where the sample with the lowest crystallinity showed the lowest melting point and enthalpy (Table II; Figures 3 and 4). Since drugs with low crystallinity could have a higher solubility, the current study suggests that it is worth measuring the crystallinity of samples after the solubility test for better interpretation of the data in the solubility test. In conclusion, the finding of this study regarding DSC and XRPD proved that if a drug is prone to any changes in its crystallinity during the solubility test, then the obtained solubility data could not be an exact reflection of the solubility data for the original crystals which was discussed further in the solubility section of the manuscript.

**Table III:** Crystallinity of Original Acetylsalicylic Acid and the Collected Acetylsalicylic Acid After the Solubility Test at Different Temperatures

Sample	25°C	37°C
Acetylsalicylic acid	44.3	44.3
100% water	32.3	23.1
100% ethanol	35.5	40.7
50:50 (water:ethanol)	33.6	42.8

### Solubility Studies

Binary aqueous-cosolvent systems are usually employed in practice to enhance the solubility of poorly water-soluble drugs in order to formulate them as a liquid dosage form for patients. Organic cosolvents particularly ethanol are among the most powerful pharmaceutical solubilizing agents which are usually used in the formulation of elixirs. The prediction of solubility trends in aqueous mixtures of ethanol is of most attention and it facilitates finding all cosolvent systems (54). The molar fraction solubility of acetylsalicylic acid in the mixed solutions with various ethanol fractions at 25 and 37°C along with standard deviations of repeated measurements are reported in Table IV. This table shows that when the volume fraction of ethanol enhances, the solubility of the drug increases and reaches a peak at the ratio of 90:10 ethanol:water at both temperatures (25 and 37°C), after which the solubility decreases. Moreover, the molar fraction solubility of acetylsalicylic acid increases when the temperature of the solution increases from 25 to 37°C at the same composition of the mixed solutions. Molar solubility value in neat water in this study at 25°C (0.0246 mol.L^−1^) and 37°C (0.0308 mol.L^−1^) is in good agreement with that reported in the literature for 25°C (0.0255 mol.L^−1^) and 37°C (0.0357 mol.L^−1^) (55) and the small observed difference can be related to the person to person error and the employed methodology. Furthermore, Table IV shows also salicylic acid concentration in the acetylsalicylic acid saturated mixtures at the investigated temperatures. The saturated concentrations of salicylic acid in the ethanol + water (56) mixtures are also reported in this table. As can be seen, salicylic acid in these mixed solutions is under saturated concentration. However, the presence of a considerable concentration of salicylic acid in the saturated solutions of acetylsalicylic acid in ethanol + water mixtures may pass some further changes in its intrinsic solubility values.

**Table IV:** Experimental Molar Fraction Solubility (*C*_*m,T*_) Values as the Mean of Three Experiments (± Standard Deviation) Measured for Acetylsalicylic Acid in Ethanol + Water Solvent Mixtures at 25 and 37°C in the Presence of Salicylic Acid (as its Degradation Product)

*f*_1_^a^	25C	37°C
Acetylsalicylic acid		
0.00	2.46 (±0.06) × 10^–2^	3.08 (±0.00) × 10^–2^
0.10	3.47 (±0.11) × 10^–2^	5.22 (±0.01) × 10^–2^
0.20	5.92 (±0.25) × 10^–2^	1.01 (±0.00) × 10^–1^
0.30	1.28 (±0.00) × 10^–1^	2.04 (±0.04) × 10^–1^
0.40	2.71 (±0.05) × 10^–1^	4.09 (±0.03) × 10^–1^
0.50	4.31 (±0.02) × 10^–1^	6.71 (±0.08) × 10^–1^
0.60	5.46 (±0.14) × 10^–1^	8.16 (±0.92) × 10^–1^
0.70	6.82 (±0.03) × 10^–1^	9.95 (±0.16) × 10^–1^
0.80	7.77 (±0.02) × 10^–1^	1.06 (±0.19)
0.90	8.68 (±0.52) × 10^–1^	1.13 (±0.08)
1.00	8.26 (±0.23) × 10^–1^	9.88 (±0.02) × 10^–1^
Salicylic acid^b^		
0.00	7.26 (±0.12) × 10^–4^ [1.37 × 10^–2^]	3.20 (±0.13) × 10^–3^
0.10	8.78 (±0.63) × 10^–4^ [1.73 × 10^–2^]	4.47 (±0.25) × 10^–3^
0.20	1.04 (±0.09) × 10^–3^ [3.24 × 10^–2^]	7.78 (±0.60) × 10^–3^
0.30	1.95 (±0.00) × 10^–3^ [8.09 × 10^–2^]	1.84 (±0.00) × 10^–2^
0.40	1.86 (±0.00) × 10^–3^ [2.10 × 10^–1^]	3.24 (±0.00) × 10^–2^
0.50	2.98 (±0.00) × 10^–3^ [4.81 × 10^–1^]	4.31 (±0.00) × 10^–2^
0.60	3.16 (±0.00) × 10^–3^ [9.62 × 10^–1^]	4.52 (±0.00) × 10^–2^
0.70	3.67 (±0.05) × 10^–3^ [1.38]	5.33 (±0.00) × 10^–2^
0.80	3.28 (±0.73) × 10^–3^ [1.84]	5.52 (±0.44) × 10^–2^
0.90	3.73 (±0.00) × 10^–3^ [2.21]	4.94 (±0.01) × 10^–2^
1.00	3.38 (±0.52) × 10^–3^ [2.11]	5.24 (±0.01) × 10^–2^

^a^*f*_1_ is volume fraction of ethanol in the ethanol and water mixtures in the absence of acetylsalicylic acid

^b^Molar concentration produced from hydrolysis of acetylsalicylic acid. Data in bracket are related to saturated concentration for salicylic acid in ethanol + water mixtures and taken from a reference (56)

**Figure 5:**
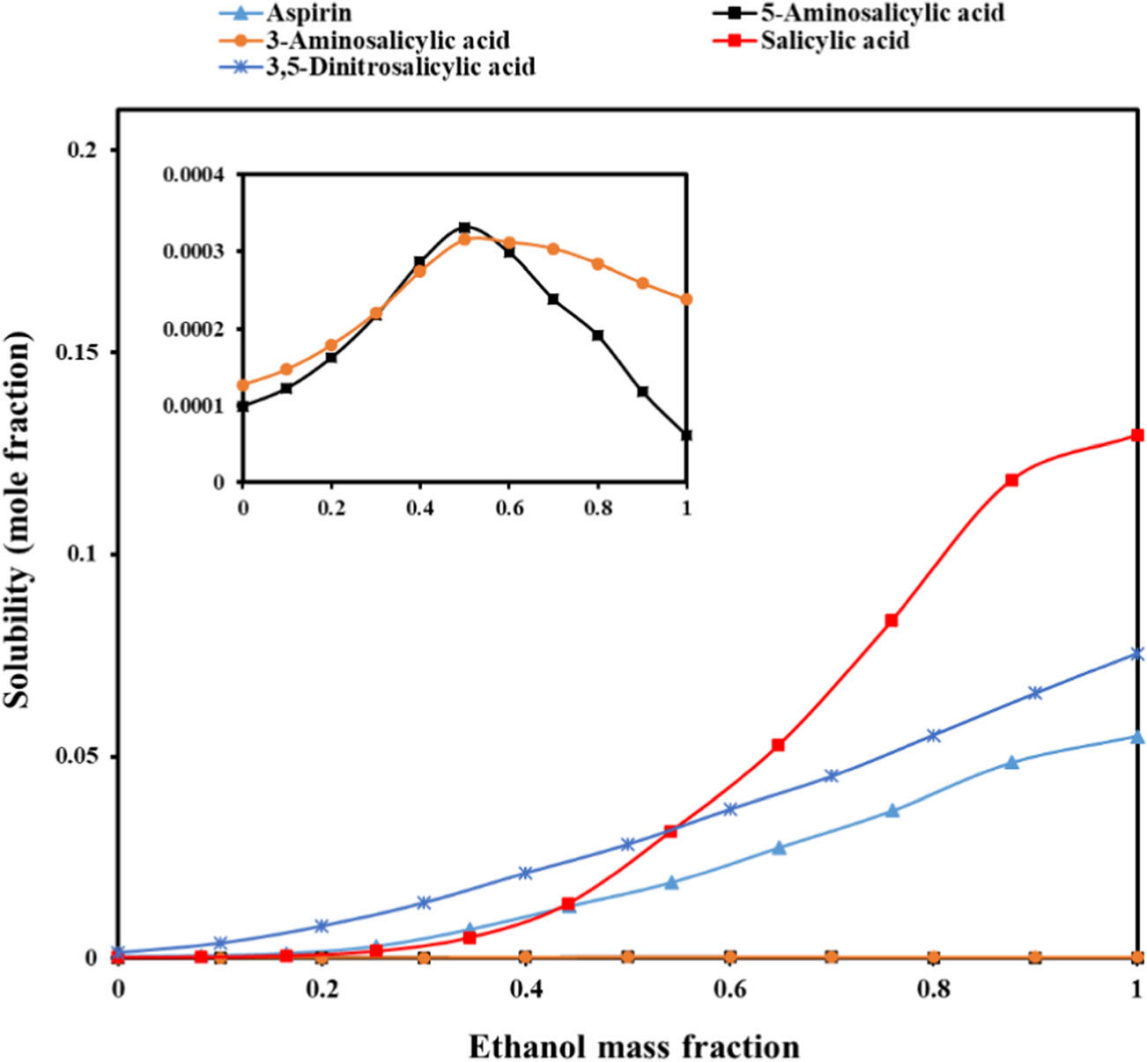
Solubility profiles of acetylsalicylic acid and other related chemicals at 25°C. The graph inside the main figure is the magnification of the solubility profiles of 3-aminosalicylic acid and 5-amino salicylic acid (these to curves are not visible in the main solubility graph)

Figure 5 compares the solubility profiles of acetylsalicylic acid (used in the current study) and other related compounds in these mixtures (i.e., salicylic acid (56), 5-amino salicylic acid (57), 3-amino salicylic acid (58), and 3,5-dinitrosalicylic acid (59)) at 25°C. As can be seen, the solubility difference between 5-amino salicylic acid and 3-amino salicylic acid is relatively small and between ethanol mass fractions of 0.6 and 1.0, and the solubility of 3-amino salicylic acid exceeded 5-amino salicylic acid. The solubility of 5-amino salicylic acid and 3-amino salicylic acid is weaker than that of acetylsalicylic acid, 3,5-dinitrosalicylic acid, and salicylic acid in the ethanol and water mixtures and shows a maximum solubility in lower ethanol mass fractions, whereas acetylsalicylic acid represents a maximum solubility at *f*_*1*_ = 0.9 (or mass fraction of 0.877) of ethanol and 3,5-dinitrosalicylic acid and salicylic acid show it at neat ethanol. For salicylic acid with log *P* of 2.26, and 3,5-dinitrosalicylic acid with log *P* of 1.71, these behaviors can be easily explained. However, for acetylsalicylic acid (log *P* of 1.18) and 5-amino salicylic acid with similar log *P* (=1.2), another parameter can affect the solubility such as steric hindrance. Generally, the solubility profile of a solute in a mixed solution is influenced by the factors of polarity, steric hindrance, hydrogen bonding, intermolecular interactions, and van der Waals’ forces between solute-solvent, solute-solute, and solvent-solvent, etc. and it is too difficult to elucidate the solubility behavior of a solute in terms of a single reason. All log *P* values were taken from DrugBank database (https://go.drugbank.com). Although the density is used for converting molar solubility data to mole fraction data, in the current study considering the instability of solute, salicylic acid also exists in the saturated solutions. So, the measured density data can not exactly be related to acetylsalicylic acid saturated solutions. However, in this section and for comparing its solubility with other salicylate derivatives, the authors used these density data for converting the molar solubility to mole fraction solubility for expressing in the same unit with other systems. It should be noted that the measured density data are considered as approximate values for acetylsalicylic acid saturated solutions considering the assumption that salicylic acid does not have a significant effect on the density value.

There is also a relationship between the aqueous solubility of a drug and its melting point. Concerning this relationship, there is no considerable change from the solubility of acetylsalicylic acid (mp = 138–140°C) to the solubility of salicylic acid (mp = 158°C); however, the aqueous solubility of 5-amino salicylic acid (mp = 283°C) is significantly lower than that of 3-amino salicylic acid (mp = 240°C).

### Mathematical Solubility Modeling

The obtained solubility data for acetylsalicylic acid in the binary mixture of ethanol + water are fitted to the equations explained in the “MATERIALS AND METHODS” section. The logarithm of solubility data or the model constants computed for the Yalkowsky model, the Jouyban-Acree, and the modified Wilson models are summarized in Tables V, VI, and VII. *MRD*% values computed for back-calculated data are also listed for each studied model. Equation 4 with *M* = 2.14 and *N* = 0.92 stated in the published research article for ethanol (60) shows an *MRD*% value of 44.5% for predicted data. Although this *MRD* is a large percentage, the need for only one datum at each employed temperature is demonstrated as the major benefit for this predictive model. The modified Wilson model provided an *MRD*% of 25.7% for the back-calculated data. The *MRD* value of < 10% for the Jouyban-Acree shows that this model can obtain precise predicted data for solubility of acetylsalicylic acid in mixtures of ethanol + water.

**Table V:** ln *C* Values of Acetylsalicylic Acid Obtained by the Yalkowsky Model (Eq. 5) in the Binary Mixture of Ethanol and Water at Different Temperatures

ln *C*		
*f*_1_	25°C	37°C
0.00	−3.71	−3.48
0.10	−3.36	−3.14
0.20	−3.02	−2.79
0.30	−2.67	−2.45
0.40	−2.33	−2.1
0.50	−1.98	−1.76
0.60	−1.64	−1.41
0.70	−1.29	−1.07
0.80	−0.95	−0.72
0.90	−0.6	−0.38
1.00	−0.26	−0.04
*MRD*%	37.6	41.0
Overall *MRD*%		44.5

**Table VI:** Parameters Calculated for the Jouyban-Acree Model for Acetylsalicylic Acid Solubility in the Binary Mixture of Ethanol and Water

	Jouyban-Acree	
Ethanol + water	*J*_*0*_	1426.596
	*J*_*1*_	515.373
	*J*_*2*_	−1084.479
*P-*value	0.05	
*MRD*%	9.9

**Table VII:** The Modified Wilson Model Parameters at the Investigated Temperatures and the *MRD*% for Back-calculated Acetylsalicylic Acid Solubility Data in the Binary Mixture of Ethanol and Water

*T* °C	λ_12_	λ_21_	*MRD*%
25	0.394	2.537	25.2
37	0.294	3.405	25.7
Overall *MRD*%			25.4

In this study, the PC-SAFT model was used to predict the solubility data for acetylsalicylic acid in the binary mixture of ethanol + water. The predicted mole fraction solubility of acetylsalicylic acid in the mixed solutions with various ethanol fractions at 25 and 37°C is reported in Table VIII. The average ***MRD***% value of < 29.65% for the PC-SAFT indicates the relatively good performance of the PC-SAFT model for predicting solubility data for acetylsalicylic acid in the binary mixture of ethanol + water by considering the fact that no experimental data are used for this model. Although the drug solubility measurement by the experimental effort is the only reliable reported method to find the drug solubility at any preferred solvent mixture, it would be costly and time-consuming procedure in most cases. In addition to predicting solubility data using the PC SAFT model, one way to overcome this problem is using a minimum number of determining solubility data for training the solubility models and prediction of other data points based on the trained model. In a previous work (13), it has been stated that the model coefficients of the Jouyban-Acree model may be computed by engaging a minimum number of data points and is capable of predicting the test data set with a satisfactory prediction error (13). In the current study, the Jouyban-Acree model is trained to employ the minimum data, i.e., solubility value in each of utilized mono-solvents at investigated temperatures and in ethanol volume fractions of 0.7, 0.5, and 0.3 at 25°C and the solubility value in other volume fractions of ethanol are predicted using the trained model which is as the following equation.

14 $$\ln {x}_{m,T}={f}_1.\ln {x}_{1,T}+{f}_2.\ln {x}_{2,T}+1153.087\frac{f_1.{f}_2}{T}+474.742\frac{f_1.{f}_2\left({f}_1-{f}_2\right)}{T}$$

**Table VIII:** Predicted Mole Fraction Solubility (${x}_{m,T}^{\boldsymbol{perd}}$) Values^a^ for Acetylsalicylic Acid in Ethanol + Water Solvent Mixtures at 25 and 37°C Using PC SAFT EOS

*f*_1_^b^	25°C	*MRD*%	37°C	*MRD*%
0.00	2.211.10^−4^	50.0	8.14.10^−4^	48.3
0.10	3.776.10^−4^	44.6	1.40.10^−3^	36.9
0.20	1.008.10^−3^	20.4	2.34.10^−3^	7.0
0.30	1.781.10^−3^	40.7	4.68.10^−3^	5.0
0.40	3.045.10^−3^	57.5	1.00.10^−2^	10.7
0.50	4.945.10^−3^	61.6	1.84.10^−2^	14.3
0.60	7.691.10^−3^	59.0	3.24.10^−2^	7.4
0.70	0.0141	48.7	5.14.10^−2^	18.4
0.80	0.0265	27.6	6.92.10^−2^	27
0.90	0.0435	10.2	8.55.10^−2^	24.4
1.00	0.0395	28.2	7.45.10^−2^	4.3
Overall *MRD*%		40.8		18.5

^a^The measured density data are considered as approximate values for acetylsalicylic acid saturated solutions and used for converting the molar data to mole fraction data with considering this assumption that as salicylic acid is not reached above the saturated solubility in these solutions, it does not have any effect on the density value

^b^*f*_1_ is volume fraction of ethanol in the ethanol and water mixtures in the absence of acetylsalicylic acid

The obtained *MRDs%* for Eq. 14 at 25 and 37°C are 11.5% and 12.0%, with an overall *MRD%* of 11.6%.

Furthermore, some trained versions of the mathematical models suggested in the published articles such as the Jouyban-Acreemodel and its combined form with Abraham solute parameters (24) are also used to forecast the acetylsalicylic acid solubility in the mixed solutions of ethanol and water with a minimum number of solubility data. The trained models are:

15 $$\ln {x}_{m,T}={f}_1.\ln {x}_{1,T}+{f}_2.\ln {x}_{2,T}+\frac{f_1.{f}_2}{T}\left[1667.856+1117.347\left({f}_1-{f}_2\right)\right]+447.7262{\left({f}_1-{f}_2\right)}^2\Big]$$

16 $$\ln {x}_{m,T}={f}_1.\ln {x}_{1,T}+{f}_2.\ln {x}_{2,T}+\frac{f_1.{f}_2}{T}\left[1286.11+825.86E+50.69S-812.89A+300.49B-684.22V\right]+\left(\frac{f_1.{f}_2\left({f}_1-{f}_2\right)}{T}\right)\left[105.18-381.77E-740.53S+1104.24A-943.10B+1906.03V\right]+\left(\frac{f_1.{f}_2{\left({f}_1-{f}_2\right)}^2}{T}\right)\left[-1137.24-786.06E+1994.90S-83.29A+399.36B-1279.27V\right]$$

None of the data points of this investigation is employed in the training of the equations listed above, and the only needed data is the solubility data in neat water and ethanol. The used *E* (the excess molar refraction), *S* (dipolarity/polarizability of the solute), *A* (the solute’s hydrogen bond acidity), *B* (the solute’s hydrogen bond basicity), and *V* (the McGowan volume of the solute) values for acetylsalicylic acid are 0.84, 1.42, 0.57, 0.77, and 1.29, respectively. These values were computed using freely available software (http://www.ufz.de/lserd) employing the SMILE code of acetylsalicylic acid. The overall *MRD* percentages for predicted data are 33.9% ± 27.0 and 16.8% ± 16.0 for Eqs. 14 and 15, respectively. In the general trained form of the Jouyban-Acree model (i.e., Eq. 14), the *J* parameters are independent of the structure of solvent, whereas all of the solute and solvents represent diverse properties such as physical/chemical stability, dielectric constant, density, solute ionization in solvent mixtures, and varied capabilities of solubilization/desolublization which considering them can be useful in the prediction capability of models. In this study, it can be seen that introducing the solubility parameters of Abraham is able to enhance the prediction capability of the model from 33.9 to 16.8%.

## CONCLUSION

In the current research, the solubility profile of acetylsalicylic acid in the binary mixtures of ethanol + water at 25 and 37°C are measured by a simple shake-flask method and the experimental data are correlated and back-calculated by Yalkowsky, Jouyban-Acree, and the modified Wilson models. The results proved that the solubility of acetylsalicylic acid at various temperatures in binary mixtures of ethanol + water can be correlated well using the Jouyban-Acree and the modified Wilson models. This is important for the pharmaceutical industry as it can save time and reduce the cost to perform the solubility test. TheJouyban-Acree-based general cosolvency models are also extended to predict solubility data of acetylsalicylic acid by considering the fact that none of the data points in the mixed solvents of this study is used in the training process of models. During the last decades, the accuracy of the cosolvency models was improved by a factor of 3–4 as it is confirmed in this work employing solubility data of acetylsalicylic acid where the prediction error of the model presented in 1998 (i.e., Eq. 4 with the prediction error of 44.5%) was reduced to 16.8% for the model presented in 2007 (i.e., Eq. 15). It is obvious that 16.8% prediction error could be well tolerated in many practical process design computations in the pharmaceutical industry. Since PC-SAFT EOS shows an acceptable function in all ranges of systems, its applicability was explored for pharmaceuticals. The results of the PC-SAFT EOS for predicting the solubility data of acetylsalicylic acid in the binary mixtures of ethanol + water at 25 and 37°C are in agreement with experimental data, suggesting the good performance of the PC-SAFT EOS for pharmaceutical purposes. For most pharmaceutical scientists, the complicated computations associated with PC-SAFT EOS are a limiting factor for its practical applications.
